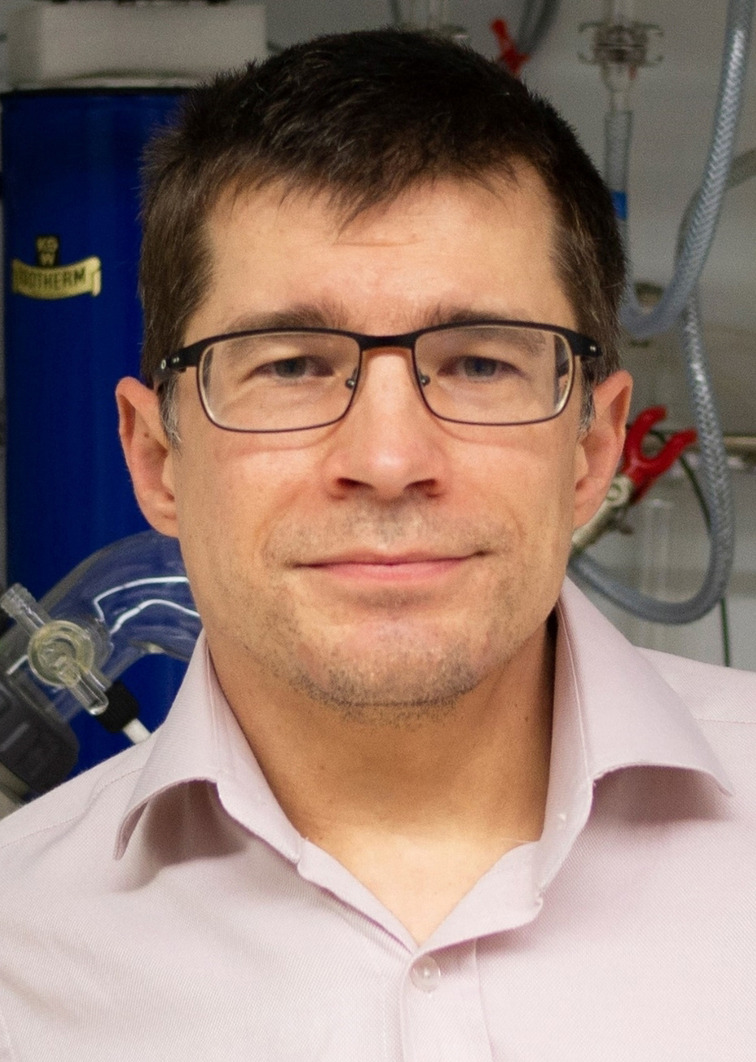# Catalytically Active Gold Nanomaterials Stabilized by *N*‐heterocyclic Carbenes

**DOI:** 10.1002/asia.202100731

**Published:** 2021-09-20

**Authors:** Constantin Eisen, Jia Min Chin, Michael R. Reithofer

**Affiliations:** ^1^ Department of Inorganic Chemistry Faculty of Chemistry University of Vienna Währinger Straße 42 1090 Vienna Austria; ^2^ Department of Physical Chemistry Faculty of Chemistry University of Vienna Währinger Straße 42 1090 Vienna Austria

**Keywords:** N-Heterocyclic Carbene, NHC, Gold nanoclusters, Gold nanoparticles, Nanoparticle catalysis

## Abstract

Solid supported or ligand capped gold nanomaterials (AuNMs) emerged as versatile and recyclable heterogeneous catalysts for a broad variety of conversions in the ongoing catalytic ′gold rush′. Existing at the border of homogeneous and heterogeneous catalysis, AuNMs offer the potential to merge high catalytic activity with significant substrate selectivity. Owing to their strong binding towards the surface atoms of AuMNs, NHCs offer tunable activation of surface atoms while maintaining selectivity and stability of the NM even under challenging conditions. This work summarizes well‐defined catalytically active NHC capped AuNMs including spherical nanoparticles and atom‐precise nanoclusters as well as the important NHC design choices towards activity and (stereo‐)selectivity enhancements.

## Graphical abstract

**NHC stabilized gold nanomaterials are emerging** as a promising material for catalysis, and this report aims to introduce them and review the current state of art.

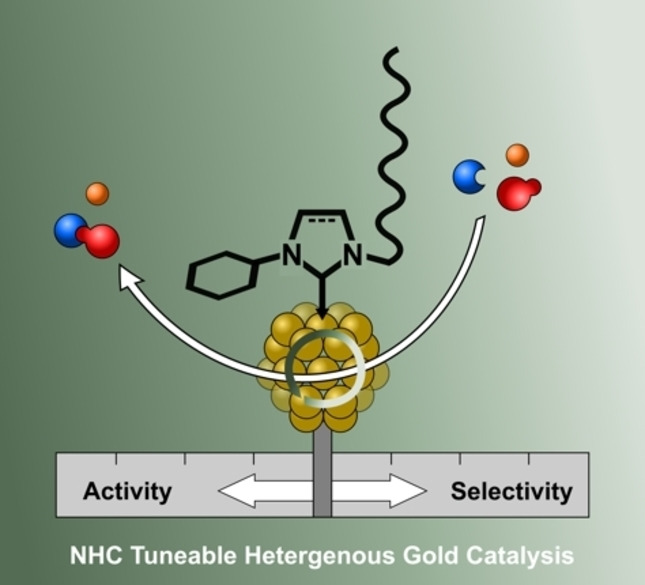

## 1 Introduction

Gold nanomaterials (AuNM) – more specifically spherical gold nanoparticles (AuNPs) – represent one of the oldest nano‐materials known to mankind and have been used for decades as colorants and in various medical applications.^[1]^ Based on the first detailed investigation of AuNPs by Faraday in 1850,^[2]^ researchers intensively worked on the characterization and size‐controlled synthesis of surface modified AuNPs^[3]^ applied in sensing,^[4]^ (bio‐) medical applications^[5]^ and catalysis.^[6]^

AuNPs benefit from a unique set of physical and material‐based properties characteristic of metallic NMs: 1) (photo‐) physical effects arising from the dense electron confinement in the gold core including quantum size effects and the color‐donating localized surface plasmon resonance (LSPR);^[7]^ 2) the formation of stable self‐assembled monolayers (SAMs) *via* a suitable ligand shell^[8]^ and 3) the high surface energy and surface‐to‐volume ratio caused by the small size of NMs which is especially beneficial for heterogeneous catalysis.^[9]^

The unique stability of elemental gold against oxidation led to its catalytic capabilities being long overlooked until examples of catalytically active AuNPs emerged in the 1970s, facilitating the successful hydrogenation of olefins,^[10]^ hydrogen and oxygen transfers between benzene/cyclohexane^[11]^ and respectively CO/CO_2_.^[12]^ This was shortly followed by Haruta's efficient oxidation of CO at low temperatures by oxide supported AuNPs.^[13]^ This breakthrough moment fueled a still lasting ′gold rush′ in catalysis.^[14]^

**Figure 1:**
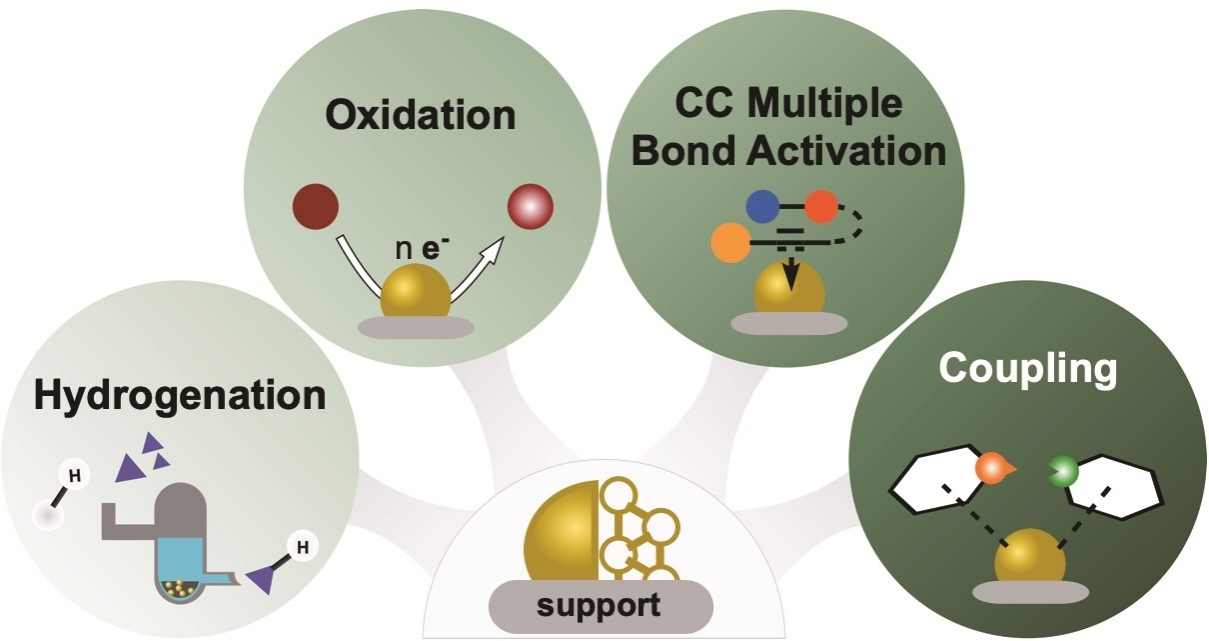
Overview on major heterogeneous conversions catalysed by AuNMCats.

The surge of gold catalysis research led to advanced (stereo‐) selective homogeneous Au(I)/Au(III) systems mainly used in the activation of CC multiple bonds^[15]^ and also highly active and substrate selective ligand capped or solid supported AuNP as recoverable heterogeneous catalysts. Today, the scope of AuNPs in catalysis ranges from selective hydrogenation and oxidation reactions over the activation of CC multiple bonds to C−C/N and multi component coupling reactions including industrial relevant examples like the hydrochloration of acetylene (Figure 1).^[16]^

**Figure 2:**
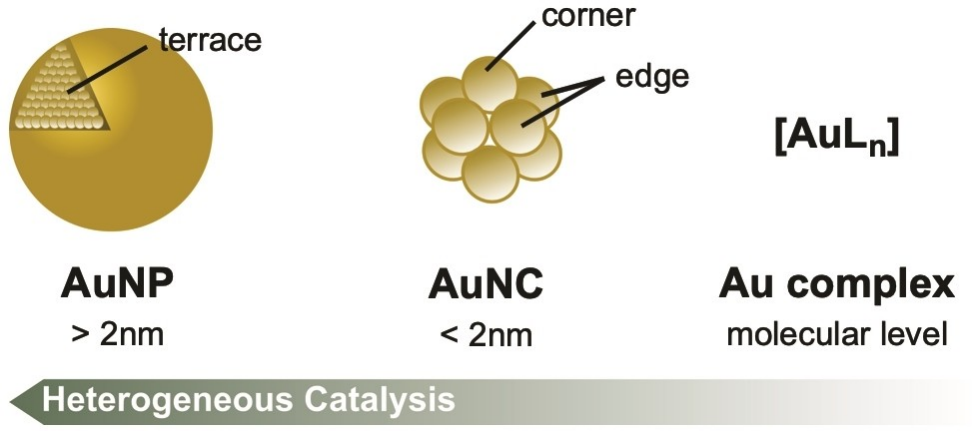
Classification of different AuNMs in terms of size and reactive sites including AuNPs, AuNCs and molecular gold complexes.

**Figure 3:**
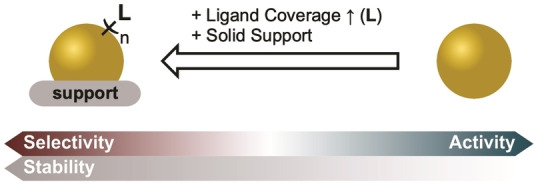
Balance of selectivity vs. activity of AuNPCats and stability gains facilitated by ligands or solid support structures.

The observed selectivity and activity of AuNP catalysts (AuNPCat) arises from the previously mentioned properties of metallic NPs but also significantly depend on shape, size, surface geometries and solid or ligand support structures.[^9^, ^17^] By having a closer look on the surface of AuNPs and atom‐precise gold clusters (AuNCs) different surface geometries are observed. While AuNPs mainly feature edge connected flat ′terrace′‐like gold surfaces, AuNCs have structures defined by variable connections of edges and corners. The different surface structures of AuNMs give rise to single gold atoms in different coordination states driving selective substrate bonding (Figure 2).[^9^, ^17f^, ^18^] High catalytic activity *via* surface‐to‐volume ratio is maintained by well separated AuNMs either through surface ligands or stabilization of AuNMs on solid supports. Stabilization of dispersed AuNPs by surface ligands causes an important consideration – the careful balance between selectivity and catalytic activity.[^17d^, ^17i^] Solid supported AuNMs without ligand coverage grant easily accessible surface atoms and therefore higher activity. As dispersed surfactant free AuNMs are thermodynamically unstable and therefore suffer from aggregation or ripening effects, a stable ligand shell needs to be employed. The ligand shell allows improved substrate attraction and (stereo‐)selectivity but lowers the activity by a decreased surface accessibility (Figure 3).[^17j^, ^19^]

To close the gap between selectivity and activity of AuNPCats, the electronic influence of ligands on gold surface atoms can be exploited as an additional handle to tune such catalysts. Through electronic surface activation by ligands, ligand‐based selectivity is maintained without loss of catalytic activity, even with increased surface coverage.[^17e^, ^17f^]

Stabilized AuNPs combine a highly interesting set of chemical and physical properties beneficial for green catalysis. Being easily recyclable by loading on support materials and stable even under harsh conditions offers a solid fundament for fine tuning selectivity and activity for a variety of catalytic applications. Targeting the balance of selectivity and activity, researchers increasingly merge the borders of organometallic and nanomaterials, leading to the introduction of strong donor ligands such as nitrogen (N) ‐heterocyclic carbenes (NHCs) to fulfill the ultimate goal in combining high activity and increased (stereo‐) selectivity in heterogeneous catalysis.^[20]^

## 2 N‐Heterocyclic Carbene Ligands for Gold Nanomaterials

Nitrogen (N) ‐heterocyclic carbenes (NHCs) are one of the most investigated class of ligands in past decades.^[21]^ Despite the low impact of early examples,^[22]^ findings by Bertrand^[23]^ and later by Arduengo^[24]^ in the stabilization and characterization of free carbenes in N‐heterocyclic systems sparked an extraordinary interest among chemists.

**Figure 4:**
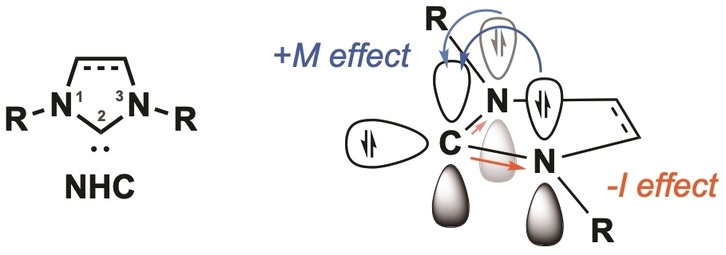
Structure of NHC and electronic stabilization of the free carbene in C2 position via the mesomeric (*+M*) and inductive (−*I*) effects.

Such NHCs offer a unique set of features including their stability and role as excellent σ‐donors in organometallic chemistry.^[25]^ The high stability of free NHCs featuring singlet carbene character arises from the electronic stabilization by the neighboring N1,3 atoms. Additionally, wingtip moieties connected to N1,3 atoms add kinetic stabilization by steric shielding of the free carbene (C2, Figure 4).^[26]^

The binding of NHCs towards Au(I) is well described and it is well known that the relativistically influenced electronics of gold play a major role in the strong connection of NHC towards gold.^[27]^ Further, the stabilized 6s and destabilized 5d orbitals of gold features a distinct sd‐hybridization which allows an ideal overlap for the electron‐rich NHC sp^2^‐orbital.[^25c^, ^28^] These electronics give rise to the 1.5‐ to 2‐fold increased stability of NHC‐Au(I) complexes compared to their phosphine and thiol counterparts.^[29]^ However, the precise bonding nature and strength of NHCs towards gold(0) surfaces is more difficult to establish. Several computational studies suggest a combination of factors contributing to the strong NHC‐Au(0) bonding. Besides the strong σ‐donor properties of NHC ligands, it is suggested that ligand surface adsorption and additional interactions of NHC wingtips contribute to the overall stability of the NHC binding to Au(0) species.^[30]^

Pairing the capability of NHCs in forming strong and tunable NHC‐metal (M) bonds with their versatile and modular synthesis,[^26^, ^31^] a broad spectrum of NHC ligands has been developed for applications in (stereo‐)selective homogeneous catalysis,^[32]^ biomedicine^[33]^ and most recently in material chemistry.^[34]^

### 2.1 NHC stabilized Gold Nanoparticles – Synthetic Aspects and Ligand Properties

By benefiting from the strong σ‐donation potential, NHCs emerged in recent years as versatile and robust surface ligand for solid‐supported and dispersed gold nanomaterials (AuNM).[^3c^, ^34^] Strongly bonded surface ligands grant good stability, which is especially important for dispersed AuNPs under harsh conditions.^[35]^ Compared to thiol stabilized AuNPs, NHC stabilized AuNPs (NHC@AuNPs), are prime candidates for future applications in biomedical^[5c]^ and catalytic applications.^[16c]^

**Scheme 1:**
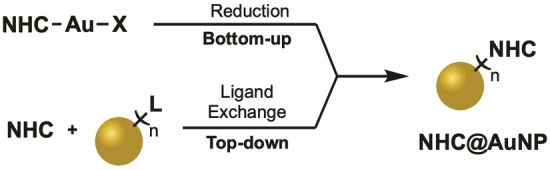
Bottom‐up and top‐down synthesis pathways of NHC@AuNPs.

The quest towards NHC@AuNPs was pioneered by Lin and coworkers with the successful reduction of gold based liquid crystals ligated by NHCs to AuNPs.^[36]^ This was shortly followed by the first targeted synthesis of NHC@AuNPs by the groups of Fairlamb/Chechik^[37]^ and Tilley^[38]^ utilizing the two common AuNP synthesis pathways – in form of the “bottom‐up” and “top‐down” strategies as illustrated in Scheme 1.

Since then, these initial reports have been followed by multiple examples demonstrating fundamental principles for attaining stable NHC@AuNPs based on detailed investigations of NHC ligand bonding,[^30a^, ^39^] synthesis^[35]^ and potential applications.

Along the strong interaction of NHCs with the surface of AuNPs, versatile synthetic toolboxes in the preparation of NHCs and their precursors allow the application focused synthesis of tailored (multi‐)functional NHC surface ligands. Within this minireview we want to boil down the key design principles leading to highly stable NHC‐functionalized AuNPs for catalytic applications.

For an general overview on existing examples of NHC@AuNP we refer to the reviews of Johnson,^[34a]^ Crudden,^[34b]^ and Glorius,^[20]^ which extensively cover fundamentals and history as well as preparation and characterization techniques of NHC stabilized NMs.

**Figure 5:**
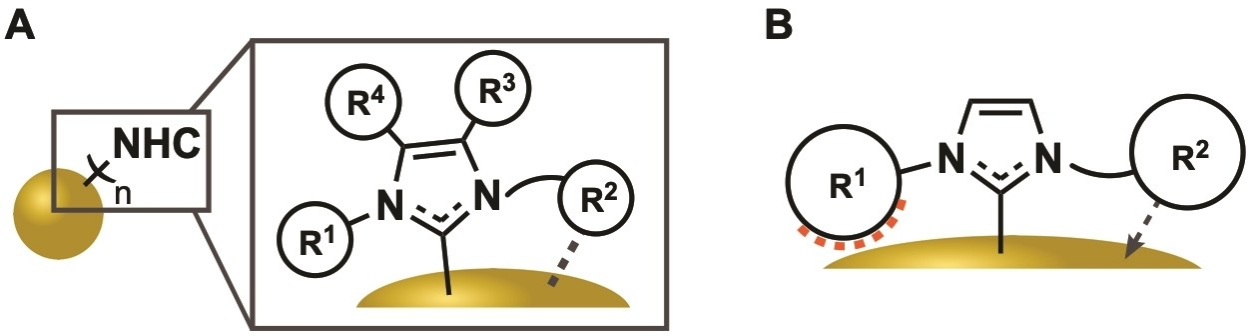
A) General schematic of an NHC attached to the Au surface; B) difference small bulky R^1^ with more rigid bulk R^2^ (steric repulsion vs additional stabilization).

By using existing synthetic NHC toolboxes, versatile functionalization in the wingtip (R^1^ and R^2^) and backbone (R^3^ and R^4^) positions[^26^, ^31^] have been achieved (Figure 5,A). Common modifications include bulky (e.g *t*Bu^[37]^ or Mes^[40]^) or flexible long‐chained groups (e. g. alkyl^[38]^ or PEG chains^[41]^) in wingtip positions. While flexible groups enhance AuNP stability in dispersion, granting narrow dispersity and allowing the assembly of superstructures, bulky NHC wingtips do not afford similar control.[^34b^, ^37^, ^38^]

**Figure 6:**
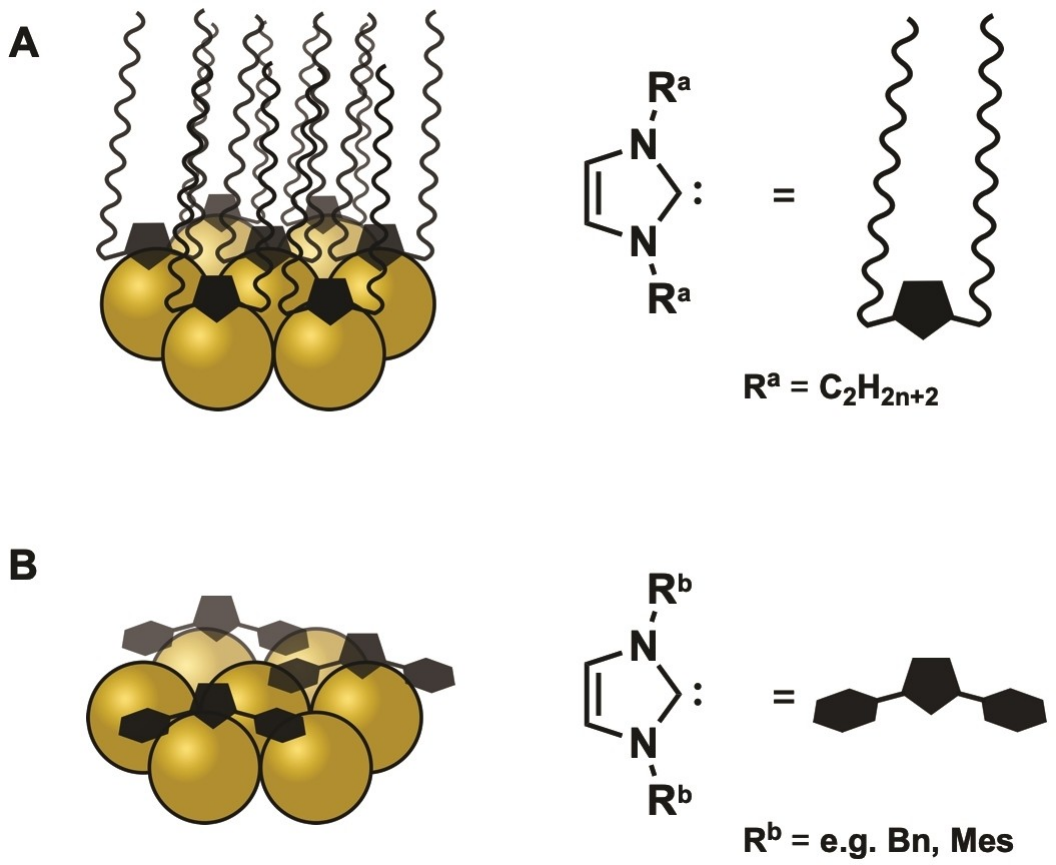
Comparison of NHC ligand packing densities based on different NHC wingtip modifications; A) NHC featuring long chains in wingtip position and B) rigid but flexible NHC wingtips.

Small bulky wingtips (e. g. *t*Bu) induce steric strain on the carbene‐AuNP bond and therefore weaken the NHC‐AuNP interaction, causing instability of obtained NHC@AuNP.^[42]^ More rigid bulky aryl wingtips on the other hand proved to be beneficial for the strength of the NHC‐AuNP interaction as well as in catalysis (Figure 6, B). Hereby, enhanced catalytic activity arises from lower ligand packing on the AuNP surface and the additional π‐interaction of aryl systems with the surface gold atoms (Figure 6, B).^[30a]^ Individual modification of R^1^ and R^2^ gives rise to asymmetric NHC ligands, allowing the introduction of charged moieties (e. g. CO_2_^−^ or SO_3_^−[43]^) and additional donor groups (e.g thiols,^[44]^ NHCs,^[45]^ aryl groups) granting additional stability *via* chelating effects. Through the introduction of a second chelating donor group, additional stability of the ligand‐AuNP interaction can be gained.[^44^, ^45^] Backbone modifications comprise long chained groups,[^42^, ^46^] charged groups^[47]^ and reactive groups for post‐modification.^[48]^ Enhanced stability of AuNPs can be further achieved by implementing NHCs in polymer chains^[49]^ and cages.^[50]^

Beyond stability of dispersed AuNPs in organic solvents, stability in aqueous media is a crucial factor for application of NHC@AuNPs in biomedical applications and in green catalysis approaches.[^5c^, ^5d^, ^51^] The necessary stability is achieved by the incorporation of hydrophilic PEG[^41^, ^46^] chains or pH‐responsive charged groups[^47^, ^52^] on the NHC.

Further, distinct chiral and asymmetric NHCs ligands are beneficial for stereoselective conversions. Nevertheless, only a few examples of AuNPs with defined chiral NHCs have been reported,^[53]^ amongst which the first fully characterized optically active NHC@AuNP was reported by our group.^[53a]^

Besides the choice of NHCs used to functionalize AuNPs, the synthetic pathway towards particle formation plays a major role in the shape, size dispersity and stability of AuNPs. While the bottom‐up strategy relies on the controlled reduction of gold containing precursors and only allows ligand‐dependent control over the final AuNPs,^[54]^ the top‐down method allows a more precise size‐ and shape‐controlled synthesis of AuNPs capped by ligands (e. g. thiols or ionic species) which can be exchanged for NHCs.^[55]^ However, the top‐down method is limited by the challenge of obtaining full ligand exchange and of thorough purification to obtain a single AuNP species. In addition, altering the surface chemistry of AuNPs can also be associated with ripening effects or aggregation of AuNPs.[^3a^, ^56^]

Furthermore, studies by Toste and by us suggest that the choice of synthetic strategy impacts the gold core of NHC‐stabilized AuNPs. Detailed XPS analyses by our group show different oxidation states of gold atoms comprised in the gold core, depending on the preparation method.[^53b^, ^57^] These results indicate different stabilities depending on the preparation method and also suggest possible decomposition pathways by leaching of NHC‐Au(I) species from the surface of AuNPs.[^30a^, ^39^, ^58^] Beside the described advantages and disadvantages both methods are readily applied in the synthesis of NHC@AuNPs.

### 2.2 NHC stabilized Gold Nanoclusters

**Figure 7:**
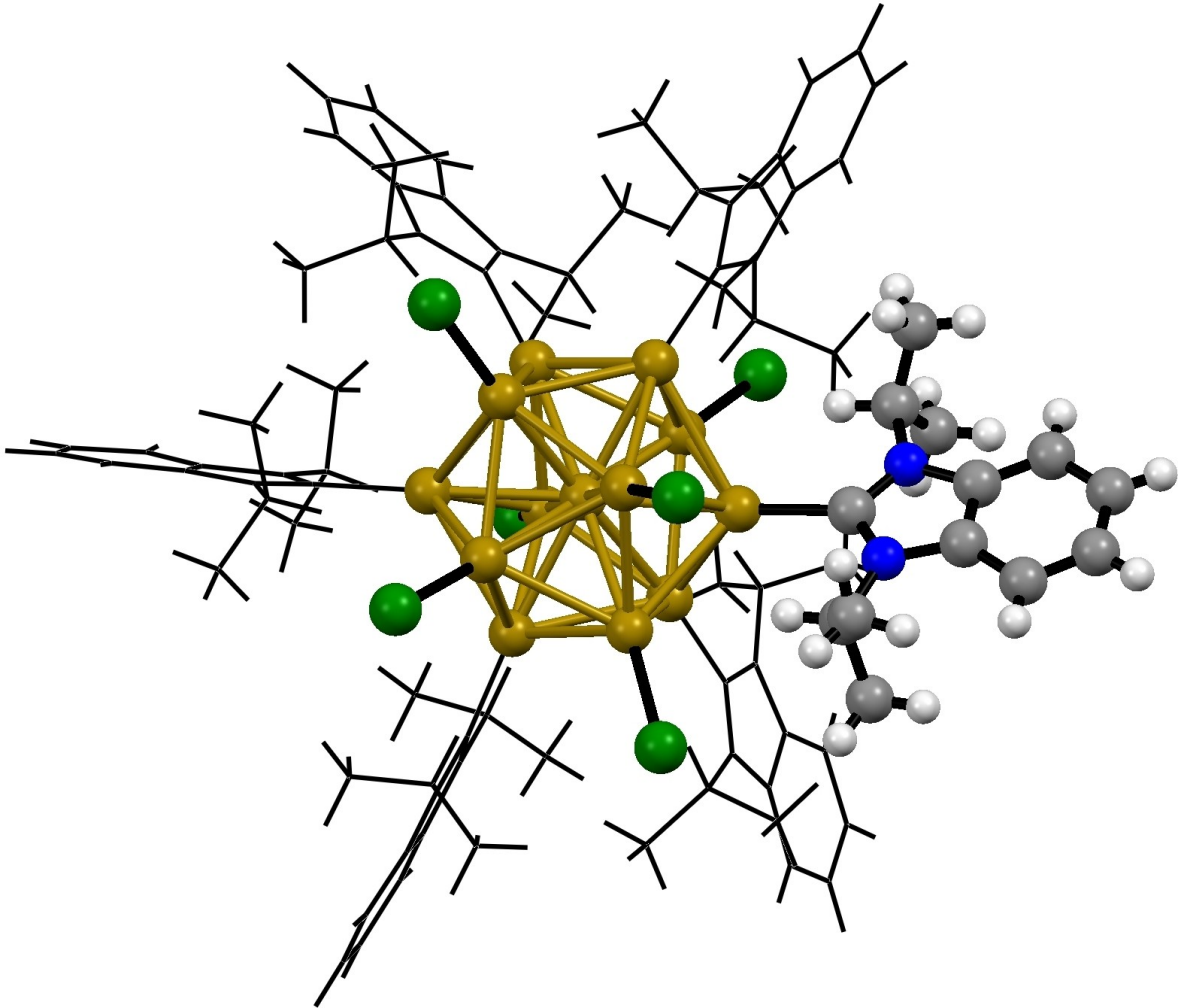
SXRD structure of [Au_13_(**NHC** ⋅ **9**)Br_6_]^−^; colour code: hydrogen (white), carbon (gray), nitrogen (blue), bromine (green) and gold (yellow); counterions and solvents are omitted for clarity.^[60]^

Atomically precise gold nanoclusters (AuNCs) occupy the gap between nano and molecular gold chemistry. AuNCs resemble ideal model systems for AuNPs by featuring true monodispersity, as well as accessibility by molecular characterization techniques such as single crystal X‐Ray diffraction (SXRD, Figure 7), thereby benefiting from well‐defined coordination chemistry and composition.^[59]^

The stability and geometric shape of AuNCs relies on certain numbers of valence electrons confined in ligand stabilized AuNCs. These specific electron numbers, which in turn arise from certain combinations of gold atoms, give rise to the ′superatom theory′ of closed electron shells by Häkkinen and coworkers and allows the explanation of so‐called “magic numbered” stable AuNCs.[^59a^, ^61^]

Similar to the development of ligand stabilized AuNPs, NHCs have been introduced as ligands for AuNCs resulting in pioneering works conducted by the groups of Crudden,^[62]^ Häkkinen and Zheng.[^60^, ^63^] Obtained AuNCs with NHC ligands show similar structure‐property relations as AuNPs and allow precise tailoring on the molecular level.

## 3 NHC stabilized Gold Nano Catalysts

NHC‐capped dispersed AuNMs offer a versatile platform for catalytic processes at the interface of heterogeneous and homogeneous gold catalysis. Their small size affords a large surface‐to‐volume ratio and increased reactivity of gold in the nanoscale,[^6a^, ^6c^] whilst NHC surface ligands grant AuNP stability in dispersion through their strong ligand‐AuNP interactions.^[34b]^ Further, the carbene‐AuNP bond is beneficial in the activation of metallic surface atoms in heterogeneous catalysis. Through the strong σ‐donation of NHCs, the surface gold atoms are electron enriched. Additional electron density leads to more Lewis basic gold atoms, increasing their nucleophilic character and allowing the fine tuning of chemoselectivity towards electron poor substrates.^[58]^ Backed by DFT calculations and comparison experiments, electronic activation by ligands has emerged as one of the main strategies for tuning of AuNMCats, alongside considerations of the coordination states of surface gold atoms and availability of reactive sites on the surface.[^6c^, ^9^, ^17b^]

In order to effectively exploit the benefits of NHC@AuNPs in heterogeneous catalysis, recyclability and stability against ripening and aggregation of used AuNPs during catalytic conversions are needed. To achieve this, catalytically active AuNM are loaded on support structures such as carbon‐ or silica‐based materials or covered in a dense ligand shell.

**Figure 8:**
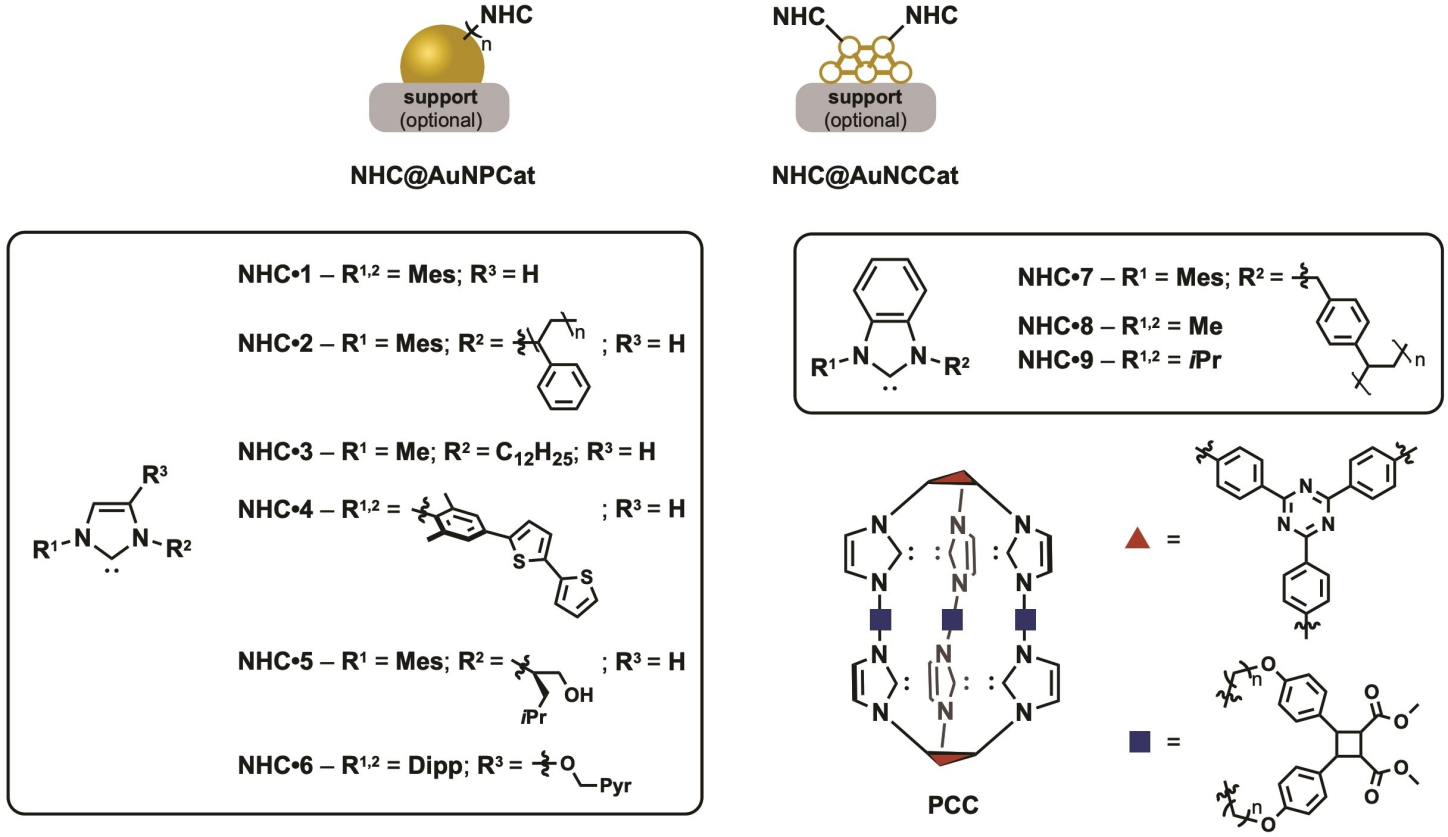
Overview on NHC ligands of catalytically active NHC@AuNP/AuNCs. Presented examples represent the most active NHC@AuNP/NC catalysts.

Similar to traditional heterogeneous gold catalysis, NHC@AuNPCat are also used in reductions and activation of CC multiple bonds.[^16c^, ^16f^] Despite the recent interest in NHC@AuNPs the scope of examples is still limited. Herein, we report some well characterized examples (Figure 8, Table 1).

**Table 1:**
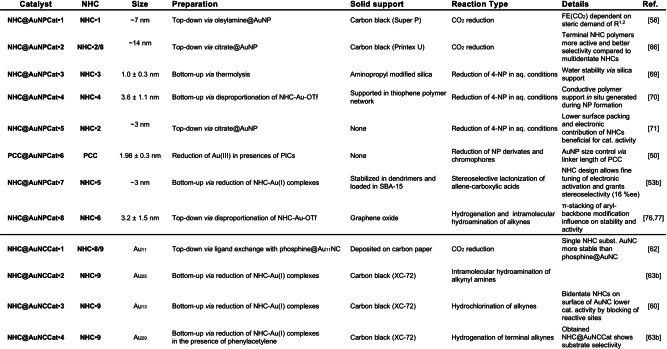
Overview on size, synthesis, NHC ligands and solid support structures of the most active NHC@AuNP/NC catalysts in their respective reference.

### 3.1 Electrochemical Reduction of CO_2_

The on‐going demand in sustainable and green catalysis led to efficient processes to convert the greenhouse gas CO_2_ into valuable organic feedstocks such as CO, methane, formate and alcohols.^[64]^ One method for carbon fixation is achieved *via* the electrochemical reduction of CO_2_ in the presence of heterogeneous AuNP catalysts.^[65]^

**Figure 9:**
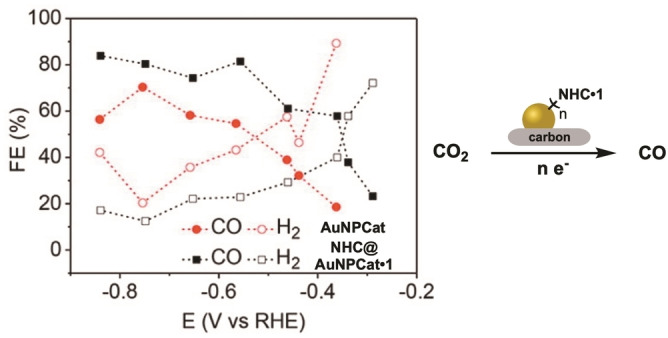
Plot of FE(%)/E(V); Comparing ligand free AuNPCat against NHC@AuNPCat ⋅ 1. Adapted with permission from Ref. [58]. Copyright (2016) American Chemical Society.

The first NHC@AuNPCat for the reduction of CO_2_ was presented by the groups of Chang and Yang in 2016. The applied catalyst is based on **NHC** ⋅ **1** (IMes) stabilized AuNPs obtained by a top‐down approach starting from oleylamine(Oa)‐capped AuNPs. The catalytically active material was achieved by loading **NHC** ⋅ **1**@AuNPs on carbon black followed by the deposition on carbon paper as working electrode in a custom two‐chambered electrical cell. Produced gaseous products were characterized by gas chromatography (GC). Obtained results show a positive onset potential of **NHC@AuNPCat** ⋅ **1** compared to bare AuNPCat and a significantly higher selectivity for CO_2_ reduction versus H_2_ formation as well as a consistently higher Faradaic efficiency for CO_2_ reduction across all potentials tested. Detailed investigations of the role of NHCs as capping ligands revealed the negative influence of increased steric bulk (*t*Bu>Dipp>Mes) on the catalytic activity in terms of the Faradaic efficiency (max. FE_CO_=83% for best cat.; Figure 9) in the formation of CO. The higher CO_2_ reduction performance of **NHC@AuNPCat** ⋅ **1** is postulated by the authors to arise from the ideal steric demand of employed NHC wingtips (Mes) which therefore enhanced CO_2_ reduction activity accordingly.^[58]^

In 2019 the groups of Liu and He reported AuNPCats capped by polymeric NHCs or polymers with terminal NHCs which were utilized for electroreduction of CO_2_. The NHC@AuNPCats were obtained by citrate exchange with CO_2_‐masked NHC ligands on AuNPs supported on carbon black. The final working electrode was obtained by drop‐casting the supported **NHC** ⋅ **2/8**@AuNPs on a pyrolytic graphite electrode and used in a two‐chambered electrical cell. Results indicate similarities to those of Chang *et al*. whereby a positive onset potential was observed while comparing **NHC@AuNPCat** ⋅ **2** with citrate@AuNPCat. Employed polymer based **NHC** ⋅ **2** ligands show enhanced selectivity towards the reduction of CO_2_ (max FE_CO_=86% for the best catalyst) over the production of H_2_ by reduction of protons. The observed selectivity was attributed to the hydrophobic nature of the NHC polymeric shell, limiting proton diffusion in aqueous conditions to the gold surface. Analysis of the ligand coverage revealed lower surface packing of multidentate polymeric **NHC** ⋅ **8** resulting in lower selectivity and surface activation compared to closer packed monodentate polymers with terminal NHCs.^[66]^

### 3.2 Reduction of Aromatic Nitro Groups

The reduction of nitro group‐containing aromatic compounds by selective hydrogenation yields derivatives of aniline which resemble valuable building blocks in the synthesis of natural compounds and pharmaceuticals.^[67]^ Gold nano catalysts (AuNMCat) in multiple compositions have shown excellent activity and selectivity in the reduction of aromatic nitro compounds with sodium borohydride in aqueous conditions. The reduction process displays an ideal model reaction due to the fast reaction progress and the ease of monitoring *via* UV‐Vis spectroscopy, which allows easy catalyst benchmarking and detailed investigation of ligand/support influences on the reactivity as well as reaction mechanisms.^[68]^

Richeter and coworkers presented in 2014 the first example of NHC@AuNPs as efficient catalysts for the reduction of 4‐nitro‐phenol (4‐NP). Thermal decomposition at 285 °C of NHC Au(I)‐C_6_F_5_ complexes resulted in ultra‐small **NHC** ⋅ **3**@AuNPs, and loading of these AuNPs on aminopropyl functionalized silica yielded in the final **NHC@AuNPCat** ⋅ **3**. The UV‐Vis monitored reaction catalyzed by **NHC@AuNPCat** ⋅ **3** (1.5 mg/mL) proceeded with a calculated rate constant, *k* of 2.3×10^−3^ s^−1^.^[69]^

In parallel, Song *et al*. showed the catalytic activity of NHC@AuNPCat supported by a conductive polymer network. The catalyst **NHC@AuNPCat** ⋅ **4** was obtained by the disproportionation of NHC‐Au(I) complexes during ligand exchange from chloride to triflate and the simultaneously triggered oxidative coupling of thiophene‐containing **NHC** ⋅ **4** wingtips to form a polymer support network. The reduction of 4‐NP with **NHC@AuNPCat** ⋅ **4** (∼2 mg/mL) was monitored by UV‐Vis which yielded a rate constant, *k*=6.29×10^−3^ s^−1^.^[70]^

He *et al*. installed NHC‐terminated polystyrene (PS) *via* ligand exchange on citrate/Oa@AuNPs to obtain the final and most active **NHC@AuNPCat** ⋅ **5**. The gold catalyzed reduction of 4‐NP was conducted with a catalyst loading of 4.9×10^−3^ mg mL^−1^ and reaction kinetics obtained by the most active **NHC@AuNPCat** ⋅ **5** show two rate constants *k_1_* =28±6×10^−3^ s^−1^ and *k*_2_=10±2×10^−3^ s^−1^. Coordination of the catalytically obtained product 4‐aminophenol to the active sites of the **NHC@AuNPCat** ⋅ **5** gives rise to the second lower rate constant *k*_2_. **NHC@AuNPCat** ⋅ **5** were benchmarked against thiols@AuNPCats and citrate@AuNPCats with similar ligand grafting densities. By comparing the catalytic activities resulting from differing surface ligands, the He group showed that AuNPs bearing **NHC** ⋅ **2** ligands outperformed those bearing thiol ligands by ∼22 times and those bearing citrate ligands by ∼5.2 times. In addition, the comparison experiments indicate the significant role of steric crowding on lowering the catalytic activity of AuNPCats.^[71]^

**Figure 10:**
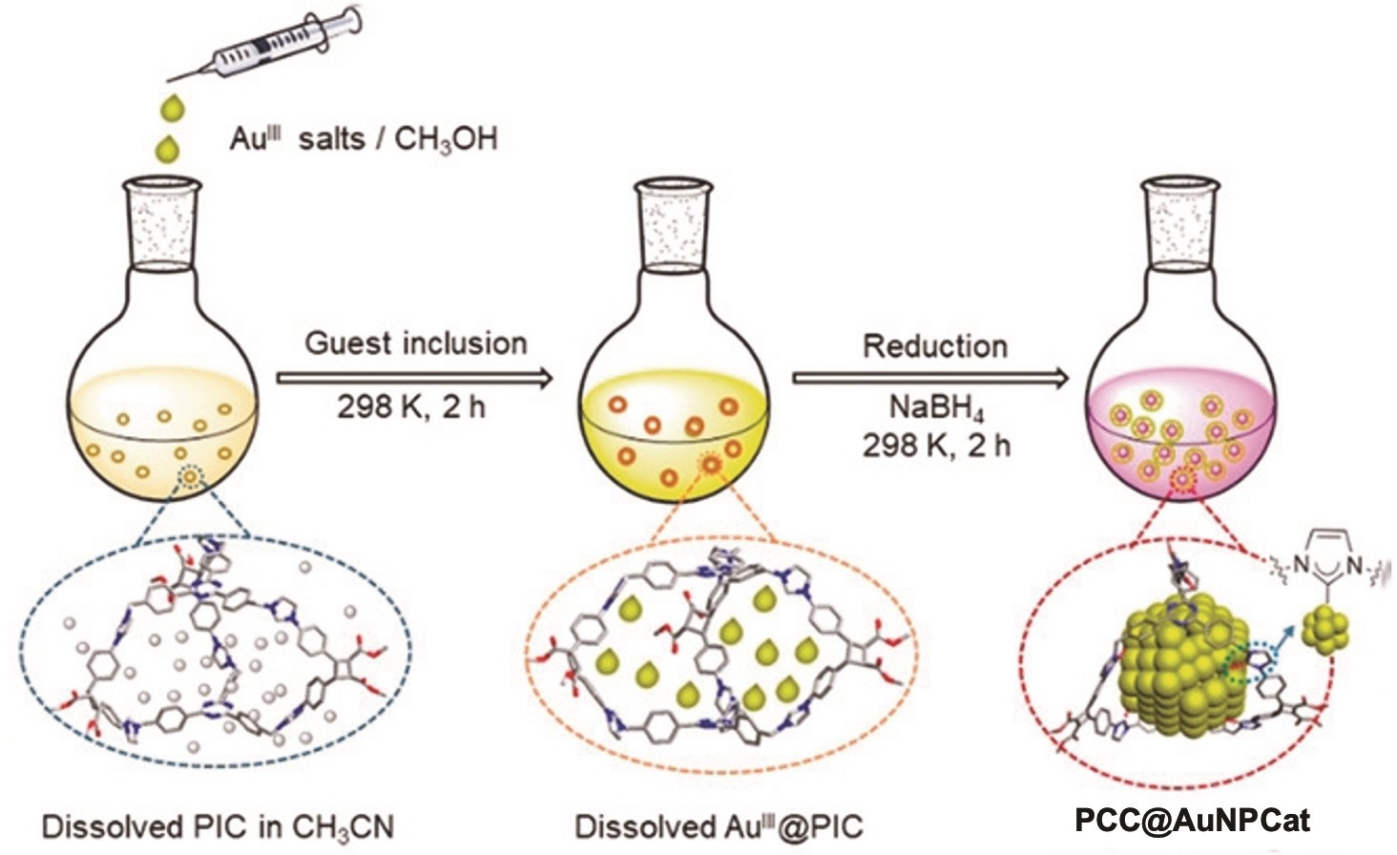
Synthetic procedure of **PCC@AuNPCats**. Adapted with permission from Ref. [50]. Copyright (2021) Wiley‐CVH.

The latest example of NHC@AuNPCat used in the reduction of aromatic nitro groups was presented by Han and coworkers in 2020. Herein, the polycarbene cages **PCC@AuNPCat** ⋅ **6** are based on AuNPs confined in polyimidazolium cages (PICs). In order to obtain the catalytically active AuNPs, Au(III) was reduced in the presence of PICs (Figure 10). Through the defined cavity volume of PCCs with linkers differing in length, robust **PCC@AuNPCat** ⋅ **6** in well‐defined sizes were synthesized. Obtained **PCC@AuNPCat** ⋅ **6** showed good catalytic activity in the reduction of 4‐NP (cat. loading 1 mg/mL) with a rate constant of *k*=2.02 min^−1^ obtained by the most active **PCC@AuNPCat** ⋅ **6**. Han and coworkers also showed successful recyclability of obtained catalysts, whereby **PCC@AuNPCat** ⋅ **6** could be used in 8 consecutive reactions without observable loss of catalytic activity. In addition, Han successfully widened the scope of reducible aromatic nitro groups to nitroanilines and the reductive splitting of azo bridges in organic dyes.^[50]^

### 3.3 Reactions Involving CC Multiple Bonds

Catalytic reactions involving compounds with CC multiple bonds – especially alkynes – resemble one of the most traditional field of homogeneous gold catalysis, with typically good catalytic activity and high selectivity.[^15b^, ^72^] With advancements in heterogeneous gold catalysis, several solid supported AuNMCats emerged in the activation of CC multiple bonds for hydration and nucleophilic additions.[^16c^, ^16f^] Example reactions include the industrially relevant hydrochlorination of acetylenes,^[16g]^ hydroaminations,^[73]^ benzannulations,^[74]^ lactonizations and multicomponent reactions like the efficient formation of different propargylamines.^[75]^

**Figure 11:**
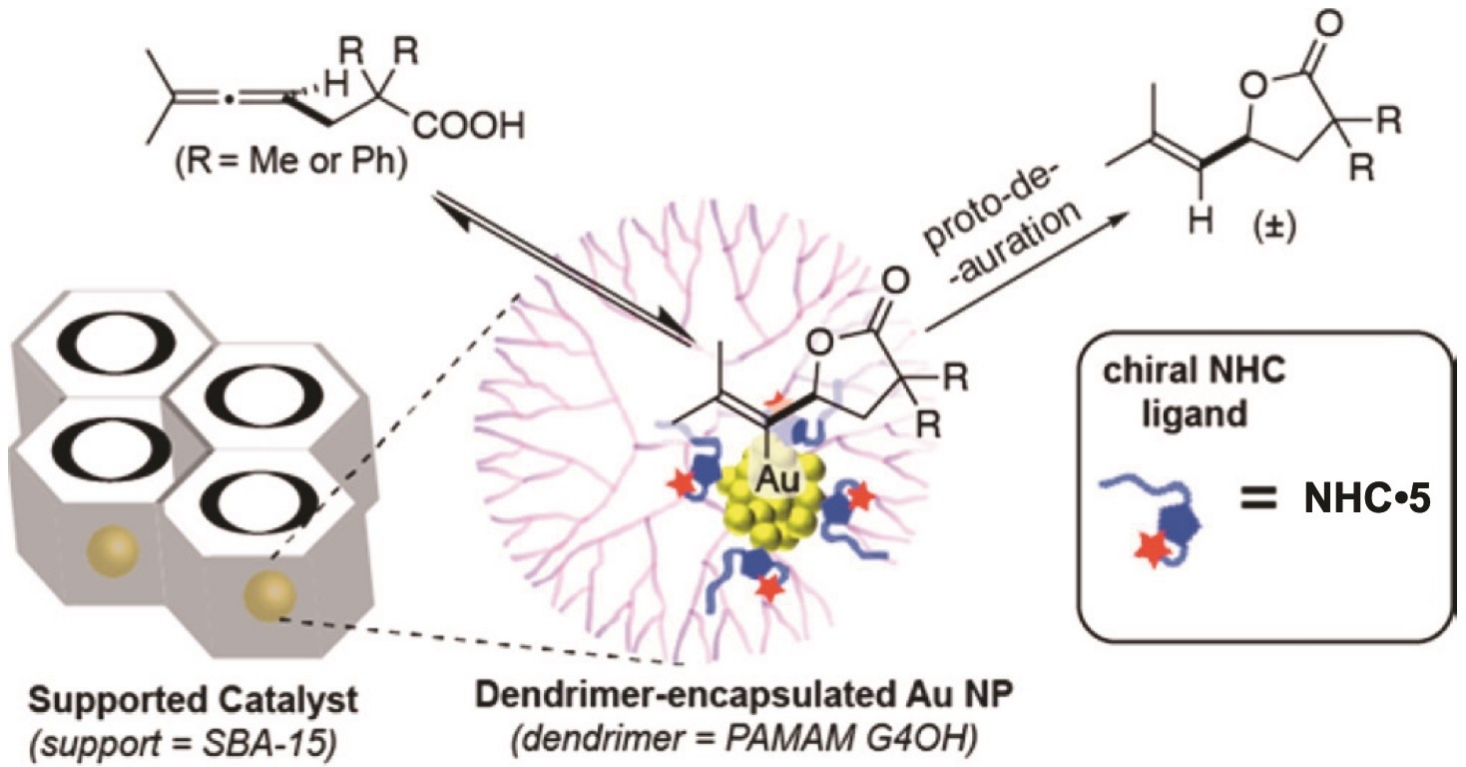
Catalyst design of **NHC@AuNPCat** ⋅ **7** and postulated reaction mechanism. Adapted with permission from Ref. [53b]. Copyright (2018) American Chemical Society.

In 2019 Toste, Somorjai and coworkers presented the first NHC@AuNPCats which allow the stereoselective lactonization of allene‐carboxylic acids. The **NHC@AuNPCat** ⋅ **7** were obtained by the reduction of chiral NHC‐Au(I) complexes with *t*Bu borane complex and subsequent stabilization of the obtained **NHC** ⋅ **5**@AuNP in dendrimer networks loaded in mesoporous silica (SBA‐15, Figure 11). **NHC@AuNPCat** ⋅ **7** allowed full substrate conversion to final lactones within 22 h at RT, while non‐NHC functionalized AuNPCats showed full conversion only at elevated temperatures. **NHC@AuNPCat** ⋅ **7** were recycled 5 times without loss in activity. Further, the asymmetric NHCs contributed to an enantiomeric excess of up to 16%. Homogeneous contributions of leached NHC‐Au(I) species were excluded by the catalytic inactivity of corresponding NHC‐Au(I) complexes under identical conditions. Obtained **NHC@AuNPCat** ⋅ **7** were investigated *via* XPS indicating a catalytically active and localized monolayer of NHC‐capped Au(I) atoms on the AuNP surface. Depending on the used NHC ligand, shifts of the Au 4f_7/2_ binding energy were observed, displaying the potential to fine tune the surface atoms of AuNPs by employing NHCs with different electronic contributions. Through the comparison of AuNPCats with differently modified NHC ligands, Toste and Somorjai also concluded the importance of structural features of used NHCs including additional activation of AuNPCats by π‐interactions of aryl groups and functional groups dictating substrate coordination, ligand rigidity for lower surface packing and asymmetry/chirality for stereoselective reactions.^[53b]^

Mata and coworkers showed NHC@AuNPCats consisting of **NHC** ⋅ **6**@AuNP loaded on graphene for the efficient hydrogenation of alkynes. The catalytically active material was obtained by the thermally driven disproportionation of NHC‐Au(I)‐OTf complexes with pyrene backbone in the presence of graphene. The **NHC@AuNPCat** ⋅ **8** allowed the efficient hydrogenation of different internal and terminal alkynes in aq. solution at 50 °C with TOFs of up to 5000 h^−1^ (1.39 s^−1^) and recyclability of the **NHC@AuNPCat** ⋅ **8** for 5 consecutive times without loss of activity. Obtained **NHC@AuNPCat** ⋅ **8** were benchmarked against the NHC‐Au(I) complexes and bare AuNP loaded on graphene. Results show the highest catalytic activity for **NHC@AuNPCat** ⋅ **8** (0.02 mol%) followed by the corresponding NHC‐Au(I) complex with 10‐fold higher catalyst loading (0.25 mol%) and then the inactive AuNPs on graphene. GC‐MS monitored filtration tests confirmed that the catalytic activity of the NHC@AuNPCat samples were of a heterogeneous nature, and not due to NHC‐Au(I) complexes. The pyrenyl‐modified backbone of NHCs used helps to stabilize the AuNPs on the graphene support by strong π‐stacking. Despite the strong interaction between NHC and the support, Mata and coworkers indicate leaching of NHC@AuNP from the support material as the potential reason for the decreasing catalytic activity after more than 5 catalytic cycles.^[76]^

**Figure 12:**
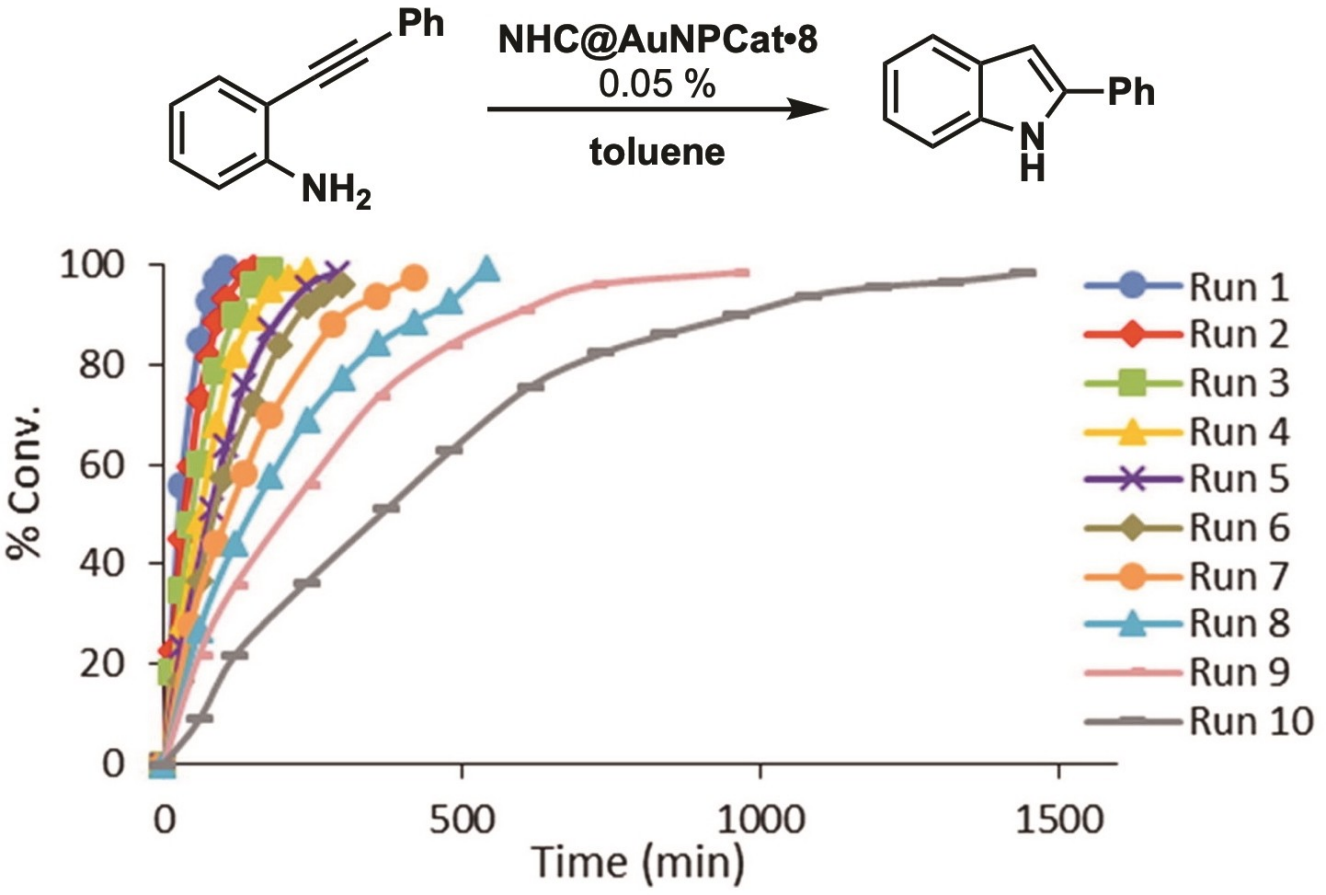
Conversion and recyclability studies of **NHC@AuNPCat** ⋅ **8**. Adapted with permission from Ref. [77]. Copyright (2020) Elsevier Inc.

In a follow up publication Mata and coworkers investigated the role of the aryl backbone modification on the catalytic activity of recycled **NHC@AuNPCat** ⋅ **8**. By decreasing the size of the aryl group, the π‐stacking towards the graphene support is lowered (pyrene (Pyr)>naphthalene (Naph)>H) and therefore a decrease in recyclability of used NHC@AuNPCats alkynes is observed. All NHC@AuNPCats were additionally tested in intramolecular hydroamination of terminal alkynes to indole derivatives in toluene at 50 °C. **NHC@AuNPCat** ⋅ **8** show good activity with low catalyst loading (0.05 mol%), full conversion with in less than 1.7 h and recyclability up to 10 times (Figure 12). Obtained results show comparable results by decreasing catalytic activity caused by gradual leaching of AuNPs from graphene underlining the importance of the solid support for NHC@AuNP used as heterogeneous catalysis.^[77]^

### 3.4 Catalytically Active NHC stabilized AuNCs

Precisely characterized ligand‐capped AuNCs allow the detailed study of ligand bonding and electronic influences, active gold sites and ultimately their catalytic activity. Occupying the gap between the molecular and nanoscale, supported AuNCs can be tailored towards active heterogeneous catalysts in common homogeneous gold catalysed reactions.^[78]^

The first example of a AuNC bearing NHC ligands was presented by Crudden and coworkers in 2019. Through the exchange of phosphine ligands by NHCs Au_11_ clusters with different levels of NHC substitution were obtained. Isolated monosubstituted [Au_11_(PPh_3_)_7_(**NHC** ⋅ **8/9**)Cl_2_]^+^ clusters (**NHC@AuNCCat** ⋅ **1**) were used in the electrochemical reduction of CO_2_ with comparable FE_CO_ of ∼80% to previously mentioned **NHC@AuNPCat** ⋅ **1/2**. Similar to AuNPCats, Crudden observed steric influences of NHC wingtips (Me>*i*Pr) and the positive electronic effects of NHCs on AuNCs in terms of catalyst activity(NHC>PR_3_).^[62]^

Häkkinen and Zheng showed the first AuNC stabilized by only NHC ligands. The isolated **NHC** ⋅ **9**@AuNC [Au_25_(**NHC** ⋅ **9**)_10_Br_7_]^2+^ were loaded on activated carbon (XC‐72) and applied as recyclable catalyst in the intramolecular hydroamination of alkynyl amines to form indoles. While Au_25_NC with thiol and phosphine ligands showed only trace conversion, **NHC@AuNCCat** ⋅ **2** showed 99% conversion within 10 h and outperformed the molecular catalyst with identical NHC ligand and catalyst loading (1.5 mol%).^[63a]^

In 2020 Zheng and coworkers synthesized a series of Au_13_NCs with NHC ligands differing in steric demand. Loading of the **NHC** ⋅ **9**@AuNC on activated carbon (XC‐72) yielded the final recyclable heterogeneous catalyst (**NHC@AuNHCat** ⋅ **3**) which facilitated the hydrochlorination of phenylacetylene at 25 °C within 4 h. Through comparison of NHC@AuNCs bearing NHC ligands of differing steric demand, Zheng and coworkers showed the importance of freely accessible gold surface. While sterically less demanding **NHC** ⋅ **9** ligands showed good catalytic activity, benzyl wingtip modified NHC@AuNCs are not active under identical conditions and catalyst loading (5 mol%).^[60]^

The latest example of NHC@AuNCCats was also presented by Häkkinen and Zheng. Using **NHC** ⋅ **9**‐capped Au_44_NC loaded on activated carbon (XC‐72) for the hydration of terminal alkynes, full conversion was obtained within 48 h at 65 °C and 8 mol% catalyst loading. Used **NHC@AuNCCat** ⋅ **4** outperformed other NHC@AuNCCat with smaller gold cores as well as molecular catalyst Au(I) catalyst with identical NHC ligands. Poisoning tests with CO confirmed the importance of accessible surface gold atoms for the catalytic activity. Further, **NHC@AuNCCat** ⋅ **4** showed substrate selectivity towards aromatic alkynes.^[63b]^

### 3.5 Additional Examples

Along fully characterized examples of NHC@AuNPCats several examples indicating the presence of NHCs as surface ligands on active AuNMCats are present in literature.

**Figure 13:**
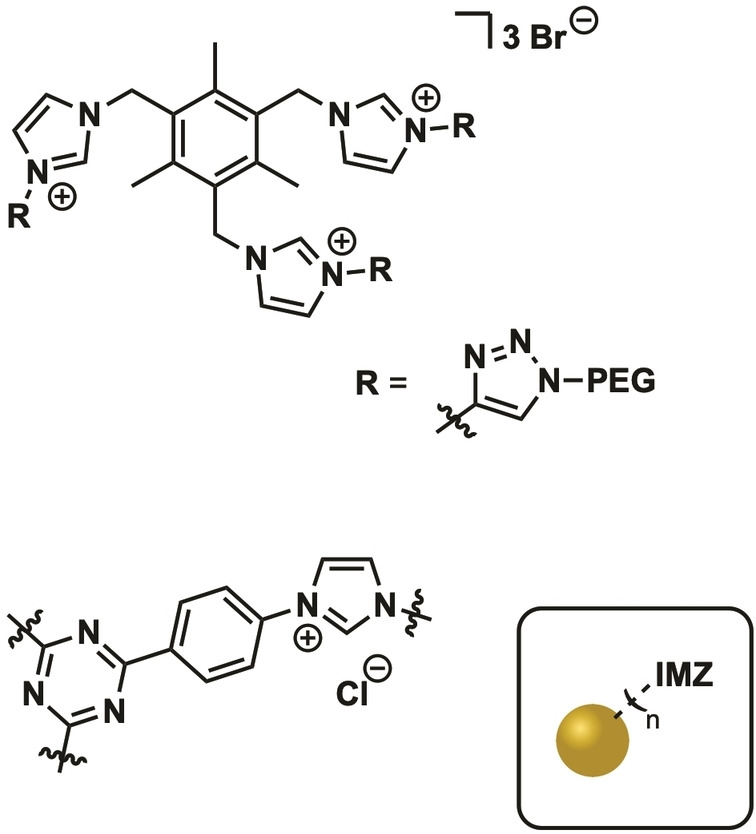
Structures of imidazolium‐based surface ligands of AuNPCats.

Pleixats and coworkers presented PEG‐tagged tris‐imidazolium salts (**IMZ** ⋅ **1**, Figure 13) as stabilizing ligands for catalytically active AuNPs in the formation of propargylamines. AuNPCats were gained by the reduction of Au(III) imidazolium salts by borane based reducing agents. Gained AuNPCats (0.5 mol%) were employed as catalysts in the reaction between aldehydes, amines and terminal alkynes to form propargylamines within 24 h at 100 °C. The most active AuNPCat was investigated in detail to understand the binding mechanism of the ligand. XPS and NMR data suggests the formation of carbene‐AuNP bonds.^[79]^

By the synthesis of imidazolium‐based cationic covalent triazine networks (ICTFs, **IMZ** ⋅ **2**) as stabilizer for AuNPs Cao *et al*. established an efficient AuNPCat for multiple successive reactions. In order to obtain the catalytically active AuNPs, Au(III) salts were reduced by sodium borohydride in the presence of ICTFs. Obtained AuNPCats feature multiple catalytically active sites, which allow a tandem conversion including deacetalization, Knoevenagl condensation and reduction to form relevant precursors for pharmaceuticals within 6 h at 60 °C and a catalyst loading of 2 mg/mL. XPS investigations of used AuNPCats indicate the presence of NHCs contributing to the strong fixation of AuNPs within the ICTF.^[80]^

## 4 Conclusion and Outlook

Despite the wide application of NHC ligands in homogeneous catalysis, catalytically active NHC stabilized metal NMs are still rare and remain in a developing stage.^[20]^ Focusing on NHC@AuNMs as catalysts, the scope is further limited to few model reactions. Nevertheless, merging the knowledge gained in the presented examples and traditional solid supported AuNMCats leads to key principles for future NHC@AuNMCats in terms of catalyst design, synthetic approaches and the crucial but still to be elucidated interplay of ligands/solid support/AuNM.

In order to achieve a catalytically active and recyclable NHC@AuNMCat, small (<5 nm) and structurally well‐defined AuNMs with suitable capping NHCs are required. The synthetic approach and design of NHC ligands have a major influence in size and shape as well as the final reactivity. Further, stabilization of obtained AuNMs on a solid support grants additional stability against ripening and aggregation even under harsh catalytic conditions.

By having a close look on the role of NHCs on the surface of AuNMCats, three principal considerations arise: 1) NHC design for strong and inert interaction between the NHC ligand and the NM surface, including a stable carbene‐AuNM bond and additional interactions *via* chelating groups; 2) appropriate electronic surface activation through the tunable σ‐donation of NHCs and 3) suitable ligand density on the surface by the variable functionalization of NHC ligands to balance catalytic stability and accessibility.

Taking the benefits of NHC modified AuNPs in consideration an outlook on the future of heterogeneous gold catalysis is possible. NHC@AuNMCats offer the unique possibility to tailor recyclable gold catalysis with improved reactivity and the simultaneous potential for improved selectivity. In addition, NHC@AuNMs suggest a significantly increased stability against harsh conditions as well as possible sintering effects and ligand leaching. The presented examples suggest the potential of NHC@AuNMCats to further close the gap between selective homogeneous and more active heterogeneous catalysis and expand the reaction scope to more complex gold‐mediated conversions.

Future developments should be focused on the mechanistic investigation of electronic surface activation by NHCs *via* precise surface characterization and computational studies to prove the suggested activation mode of AuNMCats. Further, the structural and electronic diversification of employed carbene ligands (e. g. benzimdazol‐/imidazoline‐2‐ylidenes^[81]^ and acyclic diamino carbenes^[82]^) should grant increased tunability for target‐made catalysts with increased selectivity. In these terms, NHC@AuNC are ideal model systems to benchmark different carbene ligands and establish important structure‐to‐property relations towards NHC@AuNPs.

Considering NHC@AuNMCats as green and reusable catalysts, solid supports offer the barely touched capabilities to improve stability, reactivity and selectivity *via* functionalization and structural influences. In addition, the intrinsic plasmonic effects of the gold core allow the unique light‐induced energy harvesting leading to energy‐rich electrons and localized heating beneficial for catalytic microreactor environments.

## Conflict of interest

The authors declare no conflict of interest.

## Biographical Information

Constantin Eisen completed his undergrad studies at Freie Universität Berlin (BSc.) and the University of Vienna (MSc.) with a focus on NHC chemistry towards coordination and nano chemistry. Currently, he is starting his PhD as a shared project between the University of Vienna and A*Star Singapore to on the bio‐sensing applications of ligand stabilized gold nanomaterials.

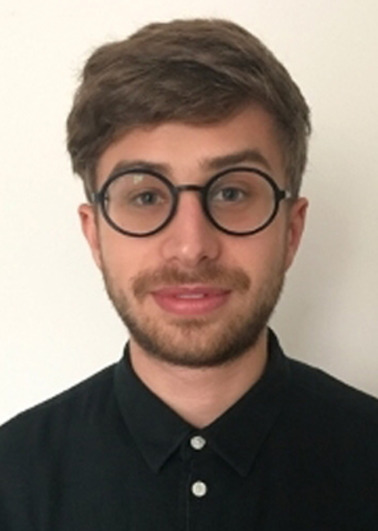

## Biographical Information

Jia Min Chin graduated from the Massachusetts Institute of Technology (MIT) after which she started as an independent researcher at the Institute of Materials Research and Engineering, A*STAR. She was then a lecturer at the University of Hull and is currently at the University of Vienna as a tenure track Professor in Physical Chemistry and a recent recipient of an ERC consolidator grant. Her work focuses on the manipulation of MOF and colloidal materials.

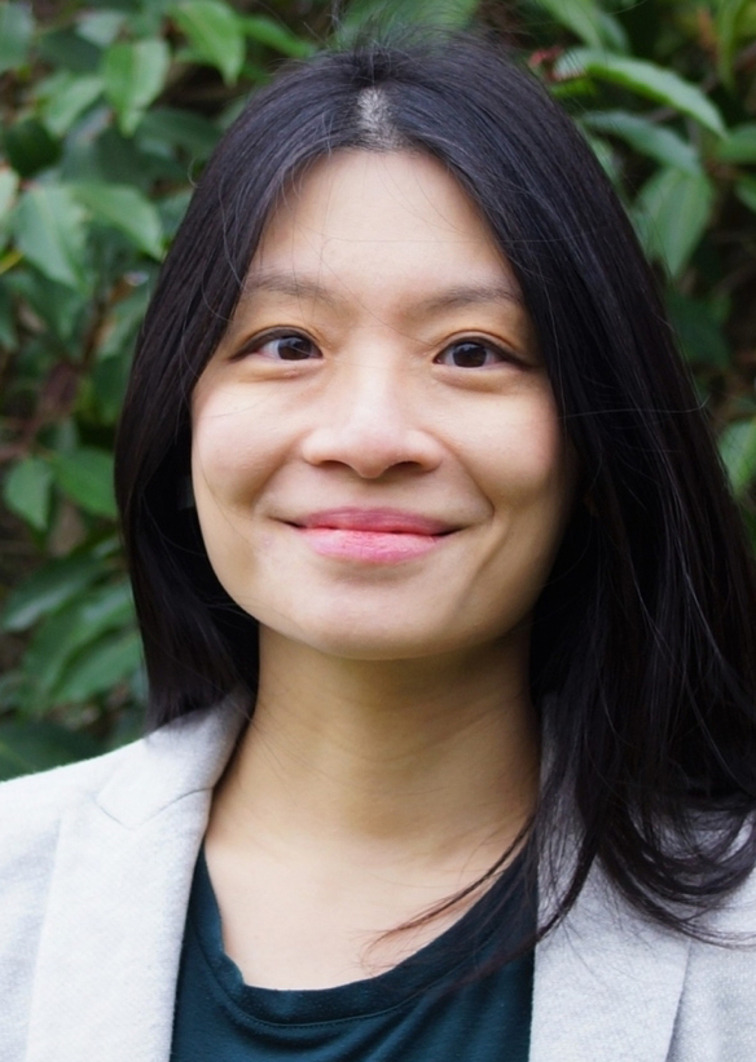

## Biographical Information

Michael R. Reithofer graduated from the University of Vienna, and performed a post‐doctoral stint at MIT on an Erwin Schrödinger Fellowship, after which he was at IBN, A*STAR as a research scientist. He started his independent career at the University of Hull in 2014 and moved to the University of Vienna where he currently is an Associate Professor in Inorganic Chemistry. His research interests include the development of metal nanomaterials for catalytic and biological applications.